# The Differentiation of Self-Motion From External Motion Is a Prerequisite for Postural Control: A Narrative Review of Visual-Vestibular Interaction

**DOI:** 10.3389/fnhum.2022.697739

**Published:** 2022-02-08

**Authors:** Shikha Chaudhary, Nicola Saywell, Denise Taylor

**Affiliations:** Rehabilitation Innovation Centre, Faculty of Health and Environmental Science, Health and Rehabilitation Research Institute, Auckland University of Technology, Auckland, New Zealand

**Keywords:** visual system, postural control, visual-vestibular interaction, visual fixations, retinal slip, optic flow, self-motion perception

## Abstract

The visual system is a source of sensory information that perceives environmental stimuli and interacts with other sensory systems to generate visual and postural responses to maintain postural stability. Although the three sensory systems; the visual, vestibular, and somatosensory systems work concurrently to maintain postural control, the visual and vestibular system interaction is vital to differentiate self-motion from external motion to maintain postural stability. The visual system influences postural control playing a key role in perceiving information required for this differentiation. The visual system’s main afferent information consists of optic flow and retinal slip that lead to the generation of visual and postural responses. Visual fixations generated by the visual system interact with the afferent information and the vestibular system to maintain visual and postural stability. This review synthesizes the roles of the visual system and their interaction with the vestibular system, to maintain postural stability.

## Introduction

Postural control requires continuous regulation of information from three systems- the visual, the vestibular, and the somatosensory (Massion, 1994; Samuel et al., 2015; Ivanenko and Gurfinkel, 2018). A key prerequisite for postural control is accurate interpretation and integration of information from the visual and vestibular systems. The interpretation and integration allow differentiation between self-motion and external motion (Redfern et al., 2001; Júnior and Barela, 2004; Guerraz and Bronstein, 2008; Rogers et al., 2017). This review focuses on fundamental concepts of the visual system and its interaction with the vestibular system required for this differentiation and will outline how it underpins efficient postural control. Postural control during conditions when vision is occluded, is not included in this review. For the purpose of this review proprioceptive information from the extraocular muscles is included as a part of the visual system, however, a more comprehensive discussion of the proprioceptive system is beyond the scope of this review. Balance is a complex function and involves multiple systems including the somatosensory, visual, and vestibular systems along with contributions from a variety of reflex control mechanisms. Whilst these are all important for postural control, this review focuses on the integration of the visual and vestibular systems.

Self-motion and motion of an object in the environment whilst a person is stationary cause a similar visual stimulation (Redfern et al., 2001; Fushiki et al., 2005; Melcher, 2011). For example, a head turn causes movement of a scene relative to the retina similar to that caused by an object’s movement within an environment, yet we perceive the environment as stationary when turning the head (Wallach, 1987; Melcher, 2011; Ivanenko and Gurfinkel, 2018). The differentiation of self-motion and external motion is essential as many everyday tasks such as walking, and driving require accurate interpretation of motion to perform each task effectively.

There are a number of reviews discussing the roles of the visual system and the vestibular system in postural control (Guerraz and Bronstein, 2008; Cullen, 2012). However, the authors of this review identified a need to synthesize key concepts of the interaction between the visual and vestibular systems. The current review outlines how this interaction underpins the differentiation of self-motion and external motion to maintain visual and postural stability.

## Overview of the Visual System

The visual system consists of the central visual system (fovea) and the peripheral visual system. The central visual system recognizes objects and object motion, whereas the peripheral vision is sensitive to moving scenes and dominates the awareness of self-motion and postural control (Dichgans and Brandt, 1978; Warren and Kurtz, 1992; Nougier et al., 1997; Berencsi et al., 2005; Guerraz and Bronstein, 2008). To maintain postural control and navigate in an environment, we need a balance between the central and peripheral vision to determine the spatial orientation of self and objects in an environment. As we move, the relationship between self, and objects in the environment changes. Accurate interpretation of these relationships using information from the visual system, helps differentiate self-motion from external motion. The following paragraph introduces three key concepts that help achieve this differentiation, optic flow, retinal slip, and visual fixations.

Optic flow is the pattern of motion of the external world over the retina and forms a part of the afferent information to the visual system (Koenderink, 1986; Warren et al., 2001; William, 2004). For example, when walking past a line of trees, there is a changing pattern of optic flow generated on the retina. Retinal slip is the movement of the visual image on the surface of the retina due to movement of the eyes and head (Strupp et al., 2003; Gielen et al., 2004; Glasauer et al., 2005). Visual fixations allow maintenance of gaze on a point and have a key role in suppressing optic flow and retinal slip, which then improves visual and postural stability (Martinez-Conde et al., 2004; Martinez-Conde, 2006; Martinez-Conde and Macknik, 2008; Otero-Millan et al., 2014). The review will focus on these three central concepts of the visual system and their interaction with the vestibular system.

## Overview of the Vestibular System

The vestibular system comprises the peripheral and central vestibular systems and serves a wide variety of functions such as postural control, gaze stabilization, conscious perception, autonomic regulation, and navigation. This review will focus on its role in postural control and gaze stabilization (Highstein et al., 2004; Tascioglu, 2005; Kanegaonkar et al., 2012; Khan and Chang, 2013; Dieterich and Brandt, 2015; Casale et al., 2020). It mediates our position in space relative to gravity and perception of self-motion by providing the sensory input to adjust position of the eye, head, and body.

The peripheral vestibular receptors provide information about the motion of the head in three dimensions. The central vestibular pathways use this information to control the reflexes and perception of self-motion (Raphan et al., 2001; Roy and Cullen, 2002; Dieterich and Brandt, 2015). The vestibulo-ocular reflex and the optokinetic reflex interact with the visual system to maintain visual and postural stability (Pettorossi et al., 1996; Kandel et al., 2000; Raphan and Cohen, 2002).

The vestibulo-ocular reflex (VOR) is a gaze stabilizing reflex which stabilizes the retinal image by rotating the eyes in the opposite direction to head movements (Paige et al., 1998; Straube, 2007; Dieterich and Brandt, 2015). It is divided into two parts: the angular VOR and the translational VOR. The angular VOR, mediated by semi-circular canals, compensates for rotational movements of the head. The translational VOR is mediated by otoliths and compensates for translation movements of the head. Gaze stabilization mediated by the VOR helps reduce optic flow and therefore retinal slip generated in response to self-motion or external motion.

Visually perceived orientation of the environment provides cues to verticality but can sometimes confound orientation. To interpret visual cues properly, the contributions of object-in-world and eye-in-world orientations from the retinal images must be reconciled to ensure an accurate perception of verticality (Sunkara et al., 2015). The vestibular system as a gravitational receptor has a fundamental role in verticality perception (Dakin and Rosenberg, 2018). This vestibular contribution to verticality perception helps to transform visual information from an eye-centered reference frame into a gravity-centered reference frame to achieve stable postural control (Dakin and Rosenberg, 2018).

## Integration

The generation of vestibular reflexes in response to visual input signifies an intimate relationship between the visual and the vestibular system such as seen in the optokinetic reflex. This reflex responds to input from the otolith organs and regulates eye position during head rotation and tilting (Mestre and Masson, 1997; Kandel et al., 2000; Tsutsumi et al., 2007). It is a combination of slow-phase and fast-phase eye movements where the eyes momentarily follow a moving object, then rapidly reset to the initial position. The optokinetic reflex is generated in response to large field movements and movement of objects in the peripheral visual field. The following sections outline visual-vestibular interactions at a functional and neuronal level.

There are three sections: (1) optic flow and postural control: this section describes how optic flow is generated, what it is used for and its role in postural control, (2) retinal slip, vestibulo-ocular reflex, and postural control: this section emphasizes how the retinal slip is interpreted and its interaction with the vestibular system to maintain postural control, (3) visual fixations and postural control: this section incorporates the role visual fixations play in postural control by interaction with the optic flow and the retinal slip. Finally, visual-vestibular interaction is discussed at the neuronal level.

### Optic Flow and Postural Control

When a person moves in an environment, it is necessary to differentiate self-motion from external motion to maintain postural stability (Wertheim, 1994; Redfern et al., 2001; Fajen and Matthis, 2013; Ramkhalawansingh et al., 2018). This distinction is dependent on perceiving whether the motion of an image on the retina is the result of a person moving relative to an object or an object moving relative to the person.

Movement of an observer in a stationary environment is interpreted as self-motion as it generates patterns of optic flow specific to self-motion (Gibson, 1950; Lappe et al., 1999; Barela et al., 2009; Fajen and Matthis, 2013). In the presence of object motion along with self-motion, the resultant optic flow is the vector sum of the object motion and self-motion components (Warren et al., 2001; Royden and Connors, 2010; Fajen and Matthis, 2013). Therefore, to achieve differentiation between self-motion and object motion, the visual system must separate the object motion component from the self-motion component. This is achieved by comparing visual information of self-motion and non-visual information of self-motion (Rushton and Warren, 2005; Guerraz and Bronstein, 2008; Royden and Connors, 2010; Fajen and Matthis, 2013). Visual information is known as retinal signal and non-visual information as the reference signal. The reference signal includes proprioceptive feedback from the extraocular muscles, the somatosensory system, vestibular afferents, and cognition. When the retinal and reference signals match, the object is perceived as stationary (the person is moving relative to the object; self-motion), when they differ, object motion is perceived (the object is moving relative to the person; object motion) (Wertheim, 1994; Wolsley et al., 1996b; Freeman, 2007; Guerraz and Bronstein, 2008; Bogadhi et al., 2013).

The optic flow pattern created during self-motion is not consistent throughout the visual field (William, 2004; DeAngelis and Angelaki, 2012). During self-motion, optic flow expands radially outwards and is projected on to the center of the retina with a focus of expansion aligned with the direction of movement, known as radial flow. In the peripheral field, optic flow remains parallel to the line of motion and sweeps past the observer, known as lamellar flow (Warren et al., 2001; Turano et al., 2005; Guerraz and Bronstein, 2008; Royden and Connors, 2010). If the object is not moving parallel to the observer, the direction of optic flow deviates from the radially expanding background flow and allows detection of the object motion during self-motion. These optic flow patterns from the environment also provide spatial-temporal information required for spatial orientation and visual navigation (Redlick et al., 2001; Warren et al., 2001; Angelaki and Hess, 2005).

In addition to optic flow, vestibular signals are important for inferring self-motion (Telford et al., 1995; Ohmi, 1996; Warren et al., 2001; Fetsch et al., 2007, 2009; Gu et al., 2008; Dokka et al., 2015). The visual and the vestibular systems have their optimal frequency ranges for providing precise cues for self-motion. The vestibular system provides information about the angular and linear acceleration of head in space, providing inputs for detecting self-motion. Information from the vestibular system is important in instances when optic flow elicits an illusion of self-motion known as vection (Brandt et al., 1972; Berthoz et al., 1975; Telford et al., 1995; Harris et al., 2000; Bertin and Berthoz, 2004). The most common real-life example of vection is, when sitting in a stationary train, movement of a neighboring train causes illusory movement of the stationary train. In such instances, a combination of information from the visual and vestibular systems is necessary to determine self-motion accurately.

### Retinal Slip, Vestibulo-Ocular Reflex, and Postural Control

Retinal slip is the afferent signal used to generate visually evoked postural reactions (Wertheim, 1994; Wolsley et al., 1996a; Guerraz and Bronstein, 2008; Lacour et al., 2018). These postural reactions’ objective is to lessen the amplitude of optic flow changes (Masson et al., 1995; Barela et al., 2009). Retinal slip is used as feedback for compensatory sway by the central nervous system (Wolsley et al., 1996a; Strupp et al., 2003; Guerraz and Bronstein, 2008).

During self-motion, objects within the visual scene move on the retina generating retinal slip, this can lead to a blurry perception of the scene and the object. To avoid this, visual and vestibular systems co-function to compensate for retinal slip by generating compensatory eye movements (Miles and Busettini, 1992; Miles, 1998; Angelaki and Hess, 2005). The eye movements comprise a vestibular driven foveal stabilization reflex known as the translational vestibular-ocular reflex (TVOR) and the visual system induced ocular following reflex (OFRs) (Miles and Wallman, 1993; Miles, 1998; Yang et al., 1999). The compensatory eye movements help maintain the target stationary on the retina while objects at different distances in the scene move relative to one another thus minimizing retinal slip (Miles and Busettini, 1992; Angelaki et al., 2003; Angelaki and Hess, 2005). The TVOR generates eye movements with an amplitude corresponding with the viewing distance (Schwarz and Miles, 1991; Angelaki and McHenry, 1999; Hess and Angelaki, 2003). The amplitude of TVOR eye movements increases as the target gets closer to the observer, enabling quick compensation for the retinal slip induced by self-motion (Angelaki and McHenry, 1999; Angelaki and Hess, 2005). The remaining retinal slip is stabilized by the ocular following reflexes (OFRs). OFRs generated in response to lamellar flow comprise conjugate vertical and horizontal eye movements. To compensate for radial flow, vergence OFRs are generated. Like TVOR, generation of OFR also depends on the viewing distance. However, TVOR dominates the compensation for first 10 milliseconds of self-motion (Schwarz and Miles, 1991; Busettini et al., 1997; Ramat and Zee, 2003).

The complexity of retinal slip increases when the observer moves closer to an object, or the object lies at an angle to the direction of motion. To maintain the body in a stable position, retinal slip must be minimized (Gielen et al., 2004). To minimize retinal slip, the amplitude of postural sway should be equal to movement of the optic flow in a direction that decreases the overall amplitude of the optic flow, which can be destabilizing for the observer (Strupp et al., 2003). To prevent destabilization, the nervous system receives information about the retinal slip by the compensatory eye movements, the TVOR, and OFR. The eye movements break down the optic flow into three components: translation, divergence, and rotational components. The disintegration minimizes the retinal slip providing cues to the central nervous system regarding the resultant retinal slip against which the compensatory postural sway is generated (Gielen et al., 2004; Angelaki and Hess, 2005). Thus, both TVOR and the OFR eliminate retinal slip maintaining visual acuity on the fovea and enabling the nervous system to provide a compensatory sway allowing the observer to maintain upright stance (Strupp et al., 2003; Angelaki and Hess, 2005).

The functioning of the VOR depends on three significant context variables; the head movement characteristics (known as stimulus context), fixation during head movements (known as fixational context), and the motion of visual target (known as visual context) (Paige, 1996; Paige et al., 1998). The head movement characteristics mainly involve the frequency and amplitude of motion. Both AVOR and LVOR operate at high frequencies (Paige et al., 1998; King and Shanidze, 2011).

For maintained fixation during head movement, VOR compensates for both translational and rotational components. Compensation is dependent on fixation distance. Fixation on a distant target requires little eye movement, as the object gets closer a larger amplitude of ocular responses is generated (Schwarz and Miles, 1991; Paige et al., 1998; Telford et al., 1998).

The mode of visual-vestibular interaction is dependent on whether the visual target is stationary or moving. If a visual target is stationary, the VOR efficiently compensates for any sudden perturbations of the head in space. Activities such as locomotion achieve gaze stability by activating semi-circular canal afferents through head movements, triggering the VOR. The eye movements generated are so accurate that there is no retinal slip, maintaining high visual acuity and gaze stability (Paige et al., 1998; Straube, 2007; Fetsch et al., 2009; Dokka et al., 2015).

### Visual Fixations and Postural Control

Visual fixations keep our eyes fixed on a target while viewing a scene. Visual fixations occur between saccades, contribute to 80% of the visual experience and are essential for visual processing (Martinez-Conde, 2006; Martinez-Conde and Macknik, 2008; Otero-Millan et al., 2014; Snodderly, 2016). Within periods of visual fixations, there are small eye movements. These small eye movements are required to overcome the neural mechanisms that lead to normalizing responses in cases of constant or uniform visual stimulation (Murakami and Cavanagh, 2001; Martinez-Conde et al., 2004; Martinez-Conde, 2006; Martinez-Conde and Macknik, 2008; Otero-Millan et al., 2012, 2014; Rucci and Poletti, 2015; Snodderly, 2016).

Visual fixations have an important role in reducing optic flow, minimizing retinal slip, and suppressing the optokinetic response (Pola et al., 1995; Glennerster et al., 2001; Murakami and Cavanagh, 2001; Uchiyama and Demura, 2009; Hoppes et al., 2018). Minimizing optic flow and retinal slip is essential as sometimes information from optic flow is destabilizing leading to generation of vection or an optokinetic response (Brandt et al., 1972; Dichgans and Brandt, 1978; Júnior and Barela, 2004; Barela et al., 2009; Dokka et al., 2015). Both instances can erroneously evoke destabilizing postural responses making a person feel unsteady and in the worst case can contribute to a fall. Interpreting information from optic flow becomes more complicated in naturalistic conditions and is significantly altered during eye and head movements and by motion of objects in the visual field (Barela et al., 2009; Fajen and Matthis, 2013; Hoppes et al., 2018). By maintaining the gaze at a single point within a scene, visual fixations increase visual stability and enhance postural control by suppressing the perception of motion within the visual field. This helps maximize the peripheral vision and provide a steady image to amplify the visual signals of self-motion (Bense et al., 2005; Martinez-Conde and Macknik, 2008; Fetsch et al., 2009; Dokka et al., 2015; Thomas et al., 2016). Sensory information from extraocular muscles then helps implementation of postural reactions (Wolsley et al., 1996b; Ivanenko and Gurfinkel, 2018).

Large field visual motion typically generates the optokinetic response (Mestre and Masson, 1997; Valmaggia and Gottlob, 2002; Tsutsumi et al., 2010). Such stimuli can lead to two interpretations; a normal one in which the observer perceives himself stationary in a moving environment or an abnormal one leading to a perception of self-motion, where moving surroundings appear stationary. Naturally, the optokinetic response is suppressed by maintaining visual fixation (Chambers and Gresty, 1982; Pola et al., 1995; Bense et al., 2005; Tsutsumi et al., 2007). Suppression of optokinetic response is required to maintain a steady image and perceive a stable world; visual-vestibular interaction is essential for visual and postural control (Bense et al., 2005; Roberts et al., 2013; Garzorz and MacNeilage, 2017). An example of this is while driving; the driver moves rapidly past stationary and moving objects, seen in the peripheral vision which would generate a rapid ocular response, if visual fixation was not able to be maintained on the road.

**FIGURE 1:**
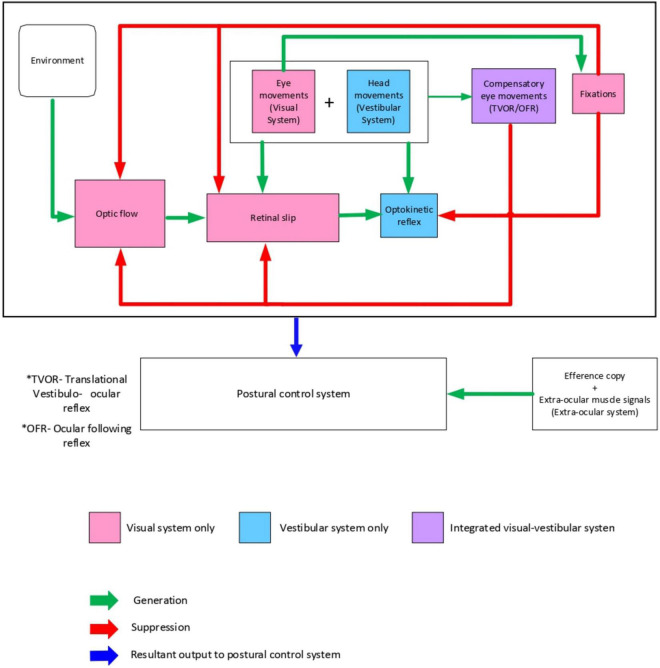
Conceptual model of visual-vestibular interaction to differentiate self-motion from external motion to maintain postural stability.

Visual fixations have a key role in maintaining postural stability as visually fixating on a target decreases postural sway (Wyatt et al., 1988, 1995; Miles and Wallman, 1993; Wallman, 1993; Uchiyama and Demura, 2009; Thomas et al., 2016; Murphy et al., 2019). Two theories have been used to explain visual fixations’ role in postural stability (Murakami and Cavanagh, 2001; Guerraz and Bronstein, 2008). The inflow theory suggests that proprioceptors in the extraocular muscles provide information about the degree of eye movements, leading to an interpretation of body shifts during postural sway. However, the outflow theory has now superseded the inflow theory. It suggests a feedforward mechanism based on the efferent copy of a motor command utilized by the central nervous system to maintain visual consistency. In this theory the magnitude of eye movements is anticipated in a feed-forward manner which provides a better explanation of what we see Figure 1.

## Neuronal Control of Visual-Vestibular Interaction

There is a large literature around neuronal control of visual-vestibular interaction. For this review, we are restricting discussion to brain areas involved in visual-vestibular interaction, neuronal mechanism for visual fixation control and interaction between these areas to maintain gaze and postural stability.

Visual-vestibular interaction is necessary to estimate and continuously update the body position in space and to distinguish self-motion from external motion. Explanation of this interaction has been widely studied in Macaque monkeys. The exact neural mechanisms for visual-vestibular integration in humans is less well understood (Roberts et al., 2017; Smith et al., 2017). Early studies have reported activation of the occipito-temporal cortex, posterior parietal cortex, and subcortical structures with reduced activation within posterior insular cortex during visual motion (Brandt et al., 1998; Dieterich et al., 1998; Kleinschmidt et al., 2002; Bense et al., 2006). Studies using caloric vestibular stimulation identified activation of similar regions with increased activity in posterior insular cortex (Bense et al., 2005, 2006).

These findings led to the current hypothesis of reciprocal visual-vestibular interaction based on reciprocal inhibition (Brandt et al., 1998). Visual-vestibular interaction depends on the pattern of visual motion as well as the active postural and locomotor tasks. This requires the nervous system to weigh out the more reliable sensory information and is known as sensory reweighting (see following for a more detailed discussion, Peterka, 2002; Assländer and Peterka, 2014). Functionally, during a constant visual input, there should be a decrease in the vestibular system’s sensitivity to head acceleration. This is essential to avoid mismatch between visual and vestibular inputs during involuntary head accelerations such as sitting facing in the opposite direction to that of the train in which you are traveling. Continuous vestibular inputs in such situations can be misleading with the perception of self-motion (Bense et al., 2005; Dokka et al., 2015). To avoid such mismatches there is a reciprocal inhibitory interaction between the visual and vestibular system (Brandt et al., 1998) where both systems suppress the other to produce a coherent sense of self-motion. Deactivation of the vestibular cortex prevents conflict between vestibular information of head motion from visually induced perception of motion and vice versa. Recent studies have identified areas of cortical activation during optic flow stimulation which are consistent with detection of self-motion (Wall and Smith, 2008; Cardin and Smith, 2010). These are regions within the intraparietal sulcus and cingulate sulcus visual area. Parieto-insular vestibular cortex and posterior insular cortex are also found to be activated during object motion (Frank et al., 2014).

A large number of areas have been associated with resolving perceptual conflicts (Nachev et al., 2008; Sharp et al., 2010; Roberts and Husain, 2015; Kolling et al., 2016). These include the insular cortex, inferior frontal gyrus, and medial frontal structures pre supplementary motor area. During conflicting visual-vestibular information there is activation of parieto- insular vestibular cortex.

Additionally, the existence of visual targets in the environment requires a combination of eye and head orientation to achieve gaze stability. The visual-vestibular interaction to shift gaze toward a target and then maintain fixation is regulated by omni-directional pause neurons (OP neurons), located in nucleus raphe interpositus of the paramedian pontine reticular formation (Prsa and Galiana, 2007; Krauzlis et al., 2017). These neurons fire during fixations and stop firing during saccades. Activity of the neurons have an inhibitory influence on saccades. They prevent firing of saccade-related premotor burst neurons which are in the mesencephalic and pontomedullary reticular formations. However, a pause in their activity allows resumption of the saccade-related burst driving the motor neurons that innervate the extraocular muscles (Krauzlis et al., 2017).

The input to the OP neurons is a weighted sum of the vestibular and visual inputs (Krauzlis et al., 2017). This comprises three signals- 1. the gaze motor error- uses a range of sensory inputs (auditory, somatosensory, and cognitive) and is the difference between the present gaze position and the final required gaze position, 2. the head velocity signal detected by the semi-circular canals by vestibular neuron and 3. the eye velocity signal. When the sum total of afferent signals surpasses a threshold the OPN’s are turned off leading to a halt in activity allowing the saccadic activity, whereas when the sum is below a threshold, OPN’s turn on and induce fixation on the target (Prsa and Galiana, 2007).

Therefore, there is a continued interaction between visual and vestibular systems for postural control to maintain body and eye stability during various transitions involving head movements and constant visual motion.

## Conclusion

The visual information regarding movements of self and objects in the environment is fundamental to postural control. Information from the optic flow patterns helps differentiate self-motion from external motion. Concurrent information of self-motion is also provided by the vestibular system using angular and linear acceleration of head in space. This information is necessary in instances when information from optic flow generates a false perception of self-motion known as vection or stimulates an optokinetic response. Optic flow patterns generate retinal slip on the retina constituting the main afferent signal to generate visually evoked postural reactions. To maintain visual and postural stability, the visual system, and the vestibular system co- function by generating TVOR and OFR’s respectively to stabilize the image on retina. Stabilization of retinal image eliminates retinal slip, providing information to the nervous system to maintain an upright stance by generating compensatory postural sway.

A key determinant of visual and postural stability is visual fixations which keep eyes fixed on a target while viewing a scene. Visual fixations suppress the optic flow and minimize retinal slip by maximizing the peripheral vision and suppressing the generation of vection. They also have a major role in suppressing the optokinetic response which can destabilize an observer. Further, they maintain visual stability during tracking a moving target by suppressing the VOR.

The current review outlines how visual-vestibular interactions enhance postural stability by interpreting the head’s position and generating eye movements accordingly, which helps differentiate self-motion or external motion and achieve gaze stabilization and postural control.

## Author Contributions

SC drafted the manuscript. DT and NS provided Ph.D. supervision for SC. All authors critically revised the manuscript read and approved the final manuscript.

## Conflict of Interest

The authors declare that the research was conducted in the absence of any commercial or financial relationships that could be construed as a potential conflict of interest.

## Publisher’s Note

All claims expressed in this article are solely those of the authors and do not necessarily represent those of their affiliated organizations, or those of the publisher, the editors and the reviewers. Any product that may be evaluated in this article, or claim that may be made by its manufacturer, is not guaranteed or endorsed by the publisher.
